# Longitudinal Characterization of the Gut Microbiota in the Diabetic ZDSD Rat Model and Therapeutic Potential of Oligofructose

**DOI:** 10.3390/metabo13050660

**Published:** 2023-05-16

**Authors:** Savanna N. Weninger, Angela Ding, Elizabeth N. Browne, Morgan L. Frost, Gabriele Schiro, Daniel Laubitz, Frank A. Duca

**Affiliations:** 1 Department of Physiological Sciences, University of Arizona, Tucson, AZ 85721, USA; 2 Department of Chemistry and Biochemistry, University of Arizona, Tucson, AZ 85721, USA; 3 School of Animal and Comparative Biomedical Sciences, College of Agricultural and Life Sciences, University of Arizona, Tucson, AZ 85721, USA; 4 The PANDA Core for Genomics and Microbiome Research, Department of Pediatrics, University of Arizona, Tucson, AZ 85721, USA; 5 BIO5 Institute, University of Arizona, Tucson, AZ 85721, USA

**Keywords:** diabetes, prediabetes, microbiome, oligofructose

## Abstract

The complex development of type 2 diabetes (T2D) creates challenges for studying the progression and treatment of the disease in animal models. A newly developed rat model of diabetes, the Zucker Diabetic Sprague Dawley (ZDSD) rat, closely parallels the progression of T2D in humans. Here, we examine the progression of T2D and associated changes in the gut microbiota in male ZDSD rats and test whether the model can be used to examine the efficacy of potential therapeutics such as prebiotics, specifically oligofructose, that target the gut microbiota. Bodyweight, adiposity, and fed/fasting blood glucose and insulin were recorded over the course of the study. Glucose and insulin tolerance tests were performed, and feces collected at 8, 16, and 24 weeks of age for short-chain fatty acids and microbiota analysis using 16s rRNA gene sequencing. At the end of 24 weeks of age, half of the rats were supplemented with 10% oligofructose and tests were repeated. We observed a transition from healthy/nondiabetic to prediabetic and overtly diabetic states, via worsened insulin and glucose tolerance and significant increases in fed/fasted glucose, followed by a significant decrease in circulating insulin. Acetate and propionate levels were significantly increased in the overt diabetic state compared to healthy and prediabetic. Microbiota analysis demonstrated alterations in the gut microbiota with shifts in alpha and beta diversity as well as alterations in specific bacterial genera in healthy compared to prediabetic and diabetic states. Oligofructose treatment improved glucose tolerance and shifted the cecal microbiota of the ZDSD rats during late-stage diabetes. These findings underscore the translational potential of ZDSD rats as a model of T2D and highlight potential gut bacteria that could impact the development of the disease or serve as a biomarker for T2D. Additionally, oligofructose treatment was able to moderately improve glucose homeostasis.

## 1. Introduction

Type 2 diabetes (T2D) is classically characterized by chronic hyperglycemia, due to increased hepatic glucose production, insulin resistance, and altered insulin production and secretion [1]. However, the development of T2D is highly complex with diet, genetics, and the environment all playing a role [2]. More recently, the gut microbiota has been implicated in the development and progression of T2D [3,4]. T2D is associated with a unique gut microbiota, and the gut microbiota has been implicated in the success of several current treatment options for diabetes [5,6]. However, the causal role of the gut microbiota in T2D is still poorly understood. For example, while inoculation of germ-free mice with the gut microbiota from diabetic mice compared to healthy mice results in impaired glucose homeostasis, this merely demonstrates the glucoregulatory impact of the gut microbiota but does not prove causality [7]. Challenges in demonstrating causality can be attributed, in part, to a lack of relevant translational models to examine T2D development in unison with alterations in the gut microbiota.

Due to the extensive limitations of studying T2D in humans, researchers have relied on animal models that mimic some of the classic features of T2D, such as increased hepatic glucose production or insulin resistance or severe loss of insulin secretion. However, current animal models for studying T2D lack the normal progression of diabetes development, do not manifest all the clinical characteristics, or have mutations in critical signaling pathways that are uncommon in humans. For example, high-fat diet (HFD)-fed mice are a commonly used model to mimic human diabetes as HFD mice are hyperglycemic, exhibit increased hepatic glucose production, and are insulin resistant. However, HFD mice lack a translational prediabetic state, and rarely develop overt diabetes with loss of insulin release and unchecked hyperglycemia [8]. The commonly used Zucker Diabetic Fatty (ZDF) rat as well as the *ob*/*ob* and *db*/*db* mouse models have genetic defects in the leptin signaling pathway, inducing obesity and insulin resistance, which are uncommon in human T2D development [9,10]. However, the Zucker Diabetic Sprague Dawley (ZDSD) rat model was recently developed as a more translational animal model for the development of diabetes. ZDSD rats are a cross between the ZDF rat and the Sprague Dawley (SD) rat, which readily develops obesity when placed on a HFD. ZDSD rats are susceptible to high fat (HF) diet-induced obesity and T2D, but unlike ZDF rats, ZDSD rats have preserved the critical leptin signaling pathway [9]. ZDSD rats have been previously characterized and present a normal progression of diabetes from 7 to 24 weeks of age, with healthy, prediabetic, and overt diabetic stages [9,11]. However, to date, no study has characterized the changes in the gut microbiota or microbial-derived short chain fatty acids (SCFAs) through the various stages of T2D in the ZDSD rat. By examining these shifts, not only can we gain a better understanding of the role of the gut microbiota in diabetes progression and discover potential therapeutic targets for T2D, but we may also uncover potential bacteria or metabolites that can serve as biomarkers for the development of T2D. In the current study, the fecal gut microbiota and fecal SCFAs were analyzed through the various stages in the progression of diabetes in the ZDSD rat.

One of the more promising gut microbiota-mediated treatments for metabolic disease is via supplementation with prebiotics, which are nondigestible food components that are selectively fermented by the gut microbiota to confer a health benefit to the host [12,13]. More specifically, the prebiotic oligofructose (OFS) beneficially shifts the gut microbiota and improves glucose and energy homeostasis in obese and diabetic rodents [14,15,16,17]. However, these studies primarily examine the effects of oligofructose in genetically modified or diet-induced obese rodents [18,19]. Therefore, we examined the effect of oligofructose in overt diabetic ZDSD rats to determine whether it could serve as a potential therapy to improve glucose homeostasis in individuals with advanced stage diabetes.

## 2. Materials and Methods

### 2.1. Animals

**Figure 1:**
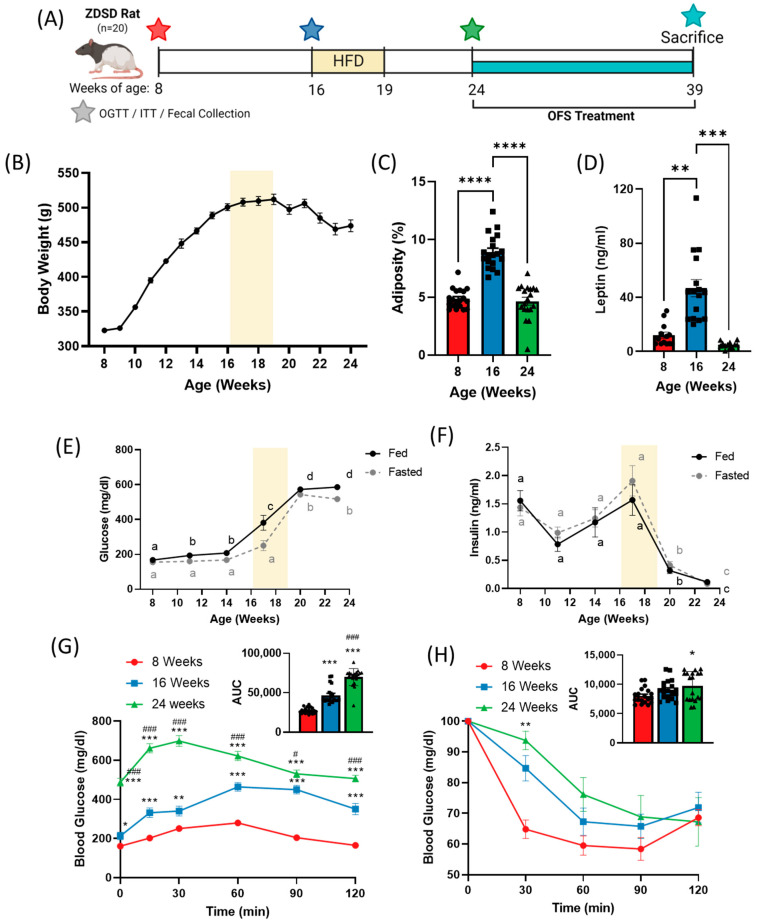
Progression of diabetes in the ZDSD rat model shifts bodyweight and adiposity and worsens glucose tolerance and insulin sensitivity. (**A**) Experimental timeline. (**B**) 24-week body weight and (**C**) adiposity and (**D**) plasma leptin at 8, 16, and 24 weeks of age in ZDSD rats on a chow diet with HF diet administered from weeks 16–19. Fed and fasted (**E**) blood glucose and (**F**) insulin over 24 weeks. (**G**) Oral glucose tolerance test and (**H**) insulin tolerance test at 8, 16, and 24 weeks. Data in all graphs represent the mean + SEM (*n* = 19); different letters represent significant differences *(p* < 0.05) over time for fed (black) or fasted (grey) glucose and insulin levels; * *p* < 0.05, ** *p* < 0.01, *** *p* < 0.001 vs. 8 weeks; **** *p* < 0.0001 between groups; # *p* < 0.05, ### *p* < 0.001 vs. 16 weeks, as assessed by two-way ANOVA with Tukey’s (**G**,**H**) or Šídák’s (**E**,**F**) multiple comparisons test or one-way ANOVA with Tukey’s multiple comparisons (**C**,**D**,**G**,**H**; AUC).

Seven-week-old male ZDSD rats were kindly provided by Charles River Laboratories. All animals were cohoused and maintained on a 12 h light/dark cycle with ad libitum access to chow (Purina 5008). At 16 weeks, rats were switched to a HFD (Research Diets D12468) for 3 weeks before being returned to their normal chow diet, in accordance with previous studies and recommended by Charles River Laboratories (personal correspondence) [9]. Body composition was measured throughout the study using quantitative magnetic resonance imaging using EchoMRI-1100 (EchoMRI, Houston, TX, USA). At the end of 24 weeks, a subset of rats was supplemented with 10% OFS in drinking water for 15 weeks (Figure 1A). The concentration of OFS in drinking water was calculated based on average amount of water and food consumed so that OFS supplementation accounted for ~10% of caloric intake.

### 2.2. Glucose and Insulin Tolerance Testing

Tolerance tests were performed at 8, 16, and 24 weeks of age, and at the end of 14 weeks of OFS treatment. For oral glucose tolerance tests, 6 h fasted rats received an oral gavage of 40% glucose solution (2 g/kg bw; Sigma G7021, St. Louis, MO, USA). Blood glucose was recorded at *t* = 0, 15, 30, 60, 90, and 120 minutes. For insulin tolerance testing, 5 h fasted rats received an intraperitoneal injection of insulin (0.75 U/kg; Sigma I0516-5ML). Blood glucose was recorded at *t* = 0, 30, 60, 90, 120 minutes.

### 2.3. Fed and Fasted Blood and Fecal Collection

Fed blood was collected at the end of the dark cycle via tail vein. Rats were then fasted for 6 h and blood collection repeated. Glucometer was used to measure glucose levels and plasma was collected and stored at −80 degrees for insulin analysis. Fresh feces were collected in cryogenic vials and snap frozen in liquid nitrogen at the 8 week, 16 week, and 24 week time points for microbiota analysis. HOMA-IR was calculated from fasting glucose and insulin (HOMA IR = (insulin (mU/L) × glucose (mg/dL))/405).

### 2.4. Tissue Collection

Prior to sacrifice, rats were fasted for 5 h and anesthetized using isoflurane. Blood from the portal vein and vena cava were collected in tubes containing DPP-IV inhibitor and plasma was stored at −20 °C. Lower small intestinal (LSI) scrapings (30 cm) were collected ending 10 cm proximal to the cecum, and colon tissue (1 cm) was collected 2 cm distal to the cecum and snap frozen in liquid nitrogen. Cecal contents were collected in cryogenic vials and snap frozen in liquid nitrogen for microbiota analysis.

### 2.5. RNA Extraction and qPCR

RNA isolation was performed using the PureLink™ RNA Mini Kit (Ambion, Austin, TX, USA) for LSI and colon tissue per the manufacturer’s protocol. cDNA was synthesized using the SuperScript™ IV VILO™ Master Mix with ezDNase™ Enzyme (Invitrogen, Waltham, MA, USA) per the manufacturer’s instruction with 3 μg of RNA. qRT-PCR was performed with CFX96 Touch™ Real-Time PCR Detection System (BioRad Laboratories, Hercules, CA, USA) using rat *Gcg* (Rn00562293_m1) and *18s* ribosomal RNA (Rn03928990_g1) TaqMan™ Gene Expression Assays (ThermoFischer Scientific, Waltham, MA, USA).

### 2.6. DNA Extraction and Microbiota Analysis

Fecal and cecal microbiota of each animal was assessed based on the V4 fragment of 16S rRNA gene as we previously performed [20,21]. The DNA extraction and library preparation were performed as we described previously [20,22]. A 7.7 pM library spiked with 10% PhiX V3 Sequencing Control (Illumina, San Diego, CA, USA) was sequenced at Microbiome Core at the University of Arizona Steele Children’s Research Center on MiSeq platform (Illumina) using custom primers [23]. The sequencing reads were demultiplexed using the *idemp* script (https://github.com/yhwu/idemp accessed on 1 April 2022). Filtering, dereplication, chimera identification, and merging of paired-end reads were performed with DADA2 [24]. The ASVs taxonomy was assigned using the Ribosomal Database Project (RDP) classifier [25] against SILVA database (release 138). Richness and Bray–Curtis dissimilarity based non-metric multidimensional scaling (NMDS) ordination were calculated using *vegan* package [26]. DeSeq2 package was used to calculate differential abundance between experimental groups. Only taxa (family and genus level) significantly different (adjusted *p* < 0.05, Wald test corrected for multiple testing using the FDR/Benjamini-Hochberg method) were presented. The Friedman test (non-parametric paired test) was used to determine the statistically significant differences between time points. Significant Friedman tests were followed by pairwise Wilcoxon signed-rank test with Bonferroni correction. Spearman correlation for metabolic parameters, SCFAs and taxa at the genus level was calculated with *cor* function form the stats package [27] and visualized with *corplot* package [28].

### 2.7. Short Chain Fatty Acid Analysis

Fecal contents were sent to Microbiome Insights for SCFA extraction as described in Zhao et al. [29]. Briefly, fecal samples were resuspended in MilliQ-grade water, and homogenized using MP Bio FastPrep, for 1 min at 4.0 m/s. Five M HCl was added to acidify fecal suspensions to a final pH of 2.0. Acidified fecal suspensions were incubated and centrifuged at 10,000 rpm to separate the supernatant. Fecal supernatants were spiked with 2-Ethylbutyric acid for a final concentration of 1 mM. Extracted SCFA supernatants were stored in 2-mL GC vials, with glass inserts. SCFAs were detected using gas chromatography (Thermo Trace 1310), coupled to a flame ionization detector (Thermo). SCFA concentrations were measured and presented per kilogram of fecal contents analyzed.

### 2.8. Biochemical Analysis

Blood glucose was measured using glucometer, unless reading exceed glucometer capabilities, in which case, plasma glucose was measured using a glucose analyzer (Analox GM9, Stourbridge, UK). Fed and fasted plasma insulin was measured via ELISA following manufacturer’s protocol (Cat. # 80-INSRT-E01, Alpco, Salem, NH, USA). Circulating leptin at 8 week, 16 week, and 24 week timepoints was measured using multiplex assay following manufacturer’s protocol (Cat. # RECYTMAG-65K, Millipore, Burlington, MA, USA). Vena cava leptin and portal glucagon-like peptide-1 (GLP-1) from control and OFS-treated rats was determined by ELISA following manufacturer’s protocol (Cat. #EGLP-35K, Millipore; Cat. # EZRL-83K, Millipore).

### 2.9. Statistical Analysis

Treatments were assigned randomly to animals and normal variance was demonstrated for all groups. One-way ANOVA with Tukey’s multiple comparisons test was used to analyze the differences between the three time points for adiposity, leptin, AUC, and SCFA levels. Two-way ANOVA with Tukey’s or Šídák’s multiple comparisons test was used to analyze fed and fasted glucose and insulin levels and glucose and insulin tolerance tests. Welch’s *t* test was used to examine differences in bodyweight, adiposity, AUC, leptin, and GLP-1 between OFS treated and untreated groups. *P* value < 0.05 was considered statistically significant.

## 3. Results

### 3.1. Bodyweight and Body Composition

The male ZDSD rat starting weight averaged 322 ± 13 g at 8 weeks of age. Bodyweight increased over 10 weeks with a peak in bodyweight at 512 ± 33 g at 19 weeks of age, followed by a decline over the next 5 weeks prior to the start of OFS treatment (Figure 1A). Adiposity, or percent fat mass, increased significantly between 8 and 16 weeks, but decreased significantly from 16 to 24 weeks (Figure 1B and Figure S1) which corresponded with drastic changes in circulating leptin (Figure 1C) and glucose homeostasis.

### 3.2. Whole Body Glucose Homeostasis

**Figure 2:**
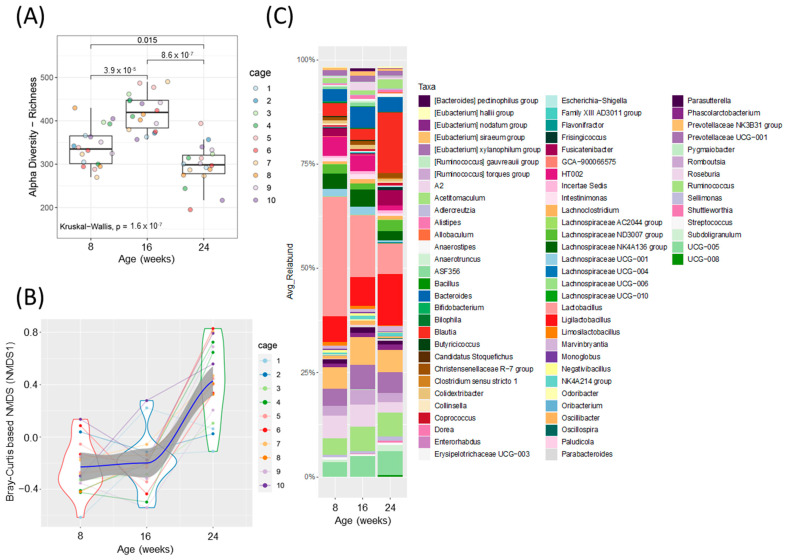
Fecal microbiota analysis throughout the development of diabetes in ZDSD rodents. (**A**) Alpha diversity index, ASV/ species richness. Statistically significant differences between groups were tested using Kruskal–Wallis test followed by pairwise Wilcoxon signed-rank test with FDR as a multiple hypothesis testing correction. (**B**) Non-metric multidimensional scaling (NMDS) using Bray–Curtis dissimilarity (NMDS 1, beta-diversity index). Each dot represents a sample from each animal at the given timepoint (disease development) and the data distribution is visualized with the kernel density violin plots with the lines connecting sample pairs from the same animal; Loess (local polynomial) regression is shown as a bold blue line with 95% confidence interval (in gray). (**C**) Taxonomic analysis at the genus level, showing relative abundance for ZDSD rodents as they progress from a healthy, to prediabetic, and diabetic state (*n* = 18, genera with relative abundance lower than 0.1% were removed for visualization only).

Over the course of the study, ZDSD rats progressed from a nondiabetic to a prediabetic followed by an overt diabetic state. Fed and fasted blood glucose significantly increased over the course of the study with a dramatic increase from 14 to 20 weeks of age (Figure 1D). Fed and fasted insulin levels peaked at 17 weeks of age before rapidly declining (Figure 1E). Oral glucose tolerance was significantly impaired at 16 weeks compared to 8 weeks of age, and further worsened at 24 weeks, as denoted by a significantly increased area under the curve (Figure 1F). Insulin tolerance tests revealed no difference in insulin tolerance at 8 and 16 weeks, but significantly decreased insulin tolerance at 24 weeks (Figure 1G). Additionally, HOMA-IR was calculated and significantly increased from 8 weeks (0.456 ± 0.208) to 16 weeks (0.774 ± 0.397; *p* = 0.067 vs. 8 weeks; *p* < 0.0001 vs. 24 weeks) before rapidly declining by 24 weeks (0.082 ± 0.082; *p* < 0.0001 vs. 16 weeks), although this is likely an artifact of dramatically reduced insulin secretion at 24 weeks (Figure 2F). Similar to previous studies [9,30], ZDSD rats established a progression of diabetes, from healthy (8 weeks) to prediabetes (16 weeks) to overt diabetes (24 weeks). Development of prediabetes was classified based on an increase in plasma glucose without a substantial change in insulin resistance, while diabetes was defined by severe hyperglycemia and impaired insulin signaling with rats exhibiting both hypoinsulinemia and insulin resistance.

### 3.3. Fecal Microbiota Analysis over Time

**Figure 3:**
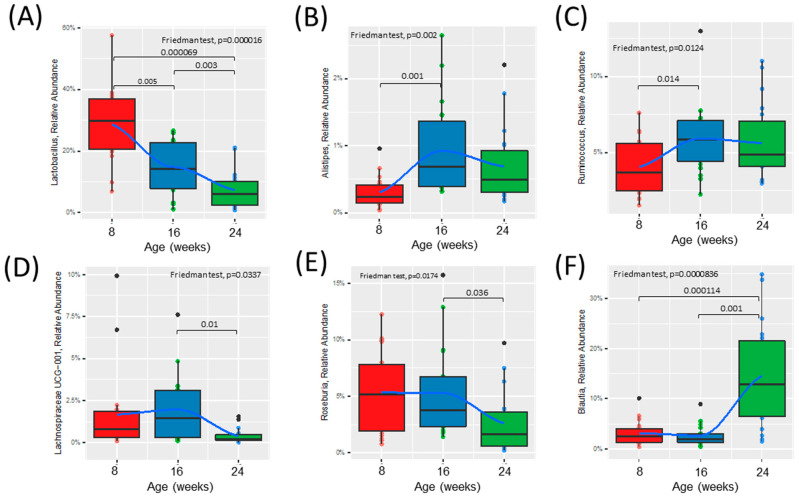
Time course analysis of the relative abundance of specific bacterial genera throughout the development of diabetes in ZDSD rodents. Presented genera were selected based on DESeq2 analysis and only taxa significantly different were chosen. (**A**–**C**) Relative abundance of bacteria significantly shifted from healthy (0) to prediabetic (8). (**D**–**F**) Relative abundance of bacteria significantly shifted from prediabetic (8) to diabetic (16) state. Statistically significant differences between groups were tested with the Friedman followed by the pairwise Wilcoxon signed-rank test with Bonferroni correction. The colored dots represent individual samples (ZDSD rats), the black dots represent outliers. The boxes visualize the median, the 25th and 75th percentile. The whiskers visualize the minimum and the maximum values. Loess (local polynomial) regression is shown as a bold blue line with 95% confidence interval (in gray).

**Figure 4:**
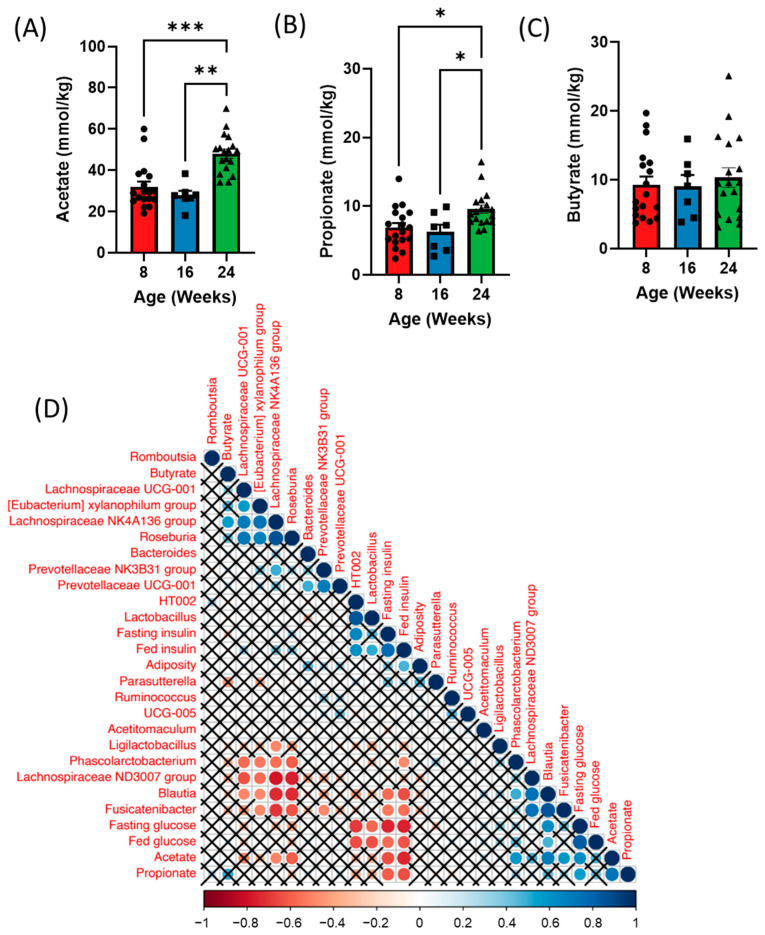
Time course analysis of fecal short chain fatty acids (SCFAs) throughout the development of diabetes in ZDSD rodents. (**A**) Acetate, (**B**) propionate, and (**C**) butyrate levels in the feces of 5 h fasted ZDSD rats. (**D**) Spearman correlation of metabolic parameters with fecal bacteria and short-chain fatty acids in ZDSD rats. Correlations that did not reach significance after correcting for False Discovery Rate are marked with an X. Data in all graphs represent the mean + SEM (*n* = 18); * *p* < 0.05, ** *p* < 0.01, *** *p* < 0.001, as assessed by Kruskal–Wallis one-way ANOVA.

Alpha diversity was increased at 16 weeks (prediabetic) compared to 8 weeks (healthy), but rapidly declined following development of diabetes at 24 weeks (diabetic) of age (Figure 2A). Beta diversity was unchanged from healthy to prediabetic states, but significantly shifted following the development of the diabetic phenotype (Figure 2B). Relative abundances of several bacterial genera shifted throughout the development of diabetes (Figure 2C and Figure S2A,B). To look more closely at the specific bacteria that were shifted between the phenotypes, we compared the relative abundance of several bacterial genera at all three timepoints (Figure 3). These specific bacterial genera were selected for time course analysis using DESeq2 analysis (Supplementary Figure S2). The genera presented in Figure 3A–C represent significant differential shifts in both the prediabetic (16 weeks of age) and diabetic (24 weeks of age), compared to healthy (8 weeks of age), thus representing changes in the microbiota that precede development of overt diabetes. The genera presented in Figure 3D–F were significantly shifted in only the diabetic state (24 weeks of age), as a representation of changes likely from the result of the diabetic phenotype. Only the bacterial genera that remained significantly shifted with the Friedman test were represented in Figure 3; all other significant changes are presented in Supplementary Figure S2. *Lactobacillus* was significantly decreased following the progression from healthy to prediabetic phenotype and further decreased following diabetes development and was negatively correlated with fed and fasting glucose and positively correlated with fed insulin (Figure 3A and Figure 4D). *Alistipes* and *Ruminococcus* were increased from healthy to prediabetes and remained elevated at 24 weeks (Figure 3B,C). These bacteria were initially shifted in the prediabetic state, indicating that they may play a causative or preventative role in the progression of diabetes. On the other hand, *Lachnospiraceae*, *Roseburia*, and *Blautia* were significantly altered only in the diabetic phenotype with reductions in *Lachnospiraceae* and *Roseburia* and increases in *Blautia* (Figure 3D–F), potentially due secondarily to host physiological changes occurring during diabetes progression. Indeed, *Blautia* was negatively correlated with fed and fasting insulin and positively correlated with fed and fasting glucose (Figure 4D).

### 3.4. Fecal Short Chain Fatty Acid (SCFA) Analysis over Time

We next examined whether shifts in the gut microbiota over time could also influence SCFA levels at each timepoint. Analysis of fecal SCFAs revealed increases in both acetate and propionate with no difference in butyrate at 24 weeks of age (diabetic) compared to both 8 (healthy) and 16 (prediabetic) weeks of age (Figure 4A–C). Additionally, we observed a significant increase in isobutyrate in the prediabetic state, and a reduction in hexanoic acid in the diabetic state (Supplementary Figure S3A–D). Both fecal acetate and propionate positively correlated with fed and fasting glucose and negatively correlated to fed and fasting insulin as well as adiposity (Figure 4D). *Blautia* was positively correlated with both acetate and propionate and negatively correlated with butyrate, while *Lachnospiraceae* and *Roseburia* were negatively correlated with acetate (Figure 4D). *Lachnospiraceae* was also positively correlated with butyrate (Figure 4D).

### 3.5. Oligofructose Increases Bodyweight and Adiposity and Improves Glucose Tolerance in Overt Diabetic ZDSD Rats

**Figure 5:**
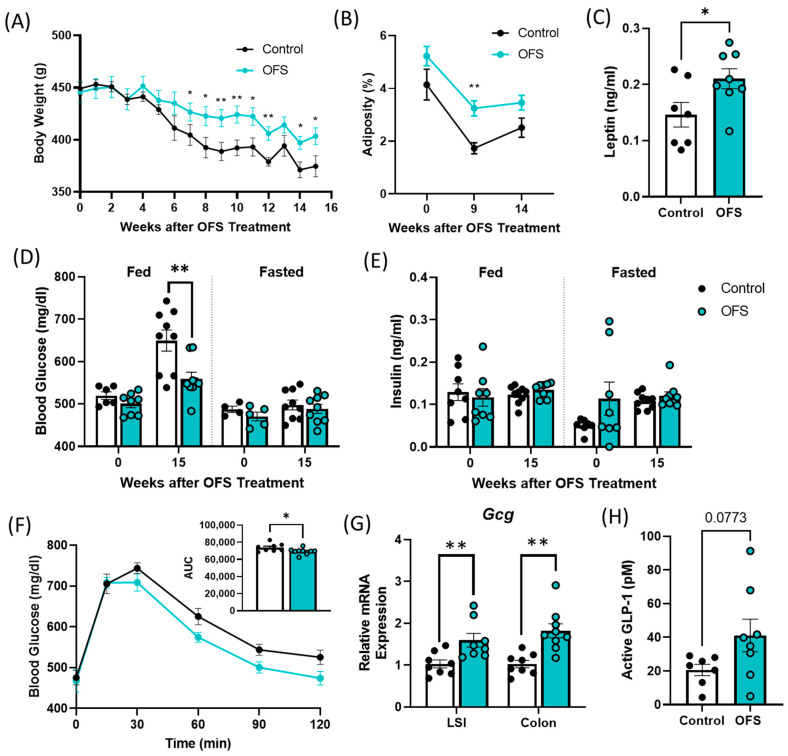
Oligofructose (OFS) increases bodyweight and adiposity and improves glucose tolerance in the ZDSD rat model of diabetes. (**A**) Bodyweight and (**B**) adiposity following supplementation with OFS in overt diabetic ZDSD rats. (**C**) Plasma leptin following 15 weeks of OFS treatment. Fed and fasted plasma, (**D**) glucose, and (**E**) insulin at baseline and following 15 weeks of OFS supplementation. (**F**) Oral glucose tolerance test following 15 weeks of OFS treatment. (**G**) Lower small intestine (LSI) and colon *Gcg* mRNA expression and (**H**) portal GLP-1 levels following 15 weeks of OFS treatment. Data in all graphs represent the mean + SEM (*n* = 18); * *p* < 0.05, ** *p* < 0.01 vs. control, as assessed by two-way ANOVA with Šídák’s multiple comparisons test or unpaired *t* test with Welch’s correction.

Supplementation of overt diabetic ZDSD rats with 10% OFS in their drinking water for 15 weeks prevented a decrease in bodyweight associated with the progression of untreated T2D. OFS-treated rats had significantly increased bodyweight compared to controls after 7 weeks of treatment and had increased adiposity after 9 weeks (Figure 5A,B and Figure S4A). In accordance with the increase in adiposity following OFS treatment, OFS-treated rats exhibited increased circulating leptin compared to controls (Figure 5C). OFS also improved oral glucose tolerance and decreased fed glucose levels with no change in insulin at the end of the study (Figure 5C–E). As OFS improves fed but not fasted glucose in overt diabetic ZDSD rats, we next measured *Gcg* mRNA in the lower small intestine (LSI) and colon. *Gcg* encodes for the incretin hormone glucagon-like peptide-1 (GLP-1), which is secreted following a meal and is known to be reduced with T2D [31]. *Gcg* expression was significantly increased with OFS treatment in both the LSI and colon of OFS-treated rats (Figure 5G). Additionally, GLP-1 was slightly increased in the portal vein following OFS treatment, although it did not reach statistical significance (Figure 5H).

### 3.6. Oligofructose Shifts the Cecal Microbiota in Overt Diabetic ZDSD Rodents

**Figure 6:**
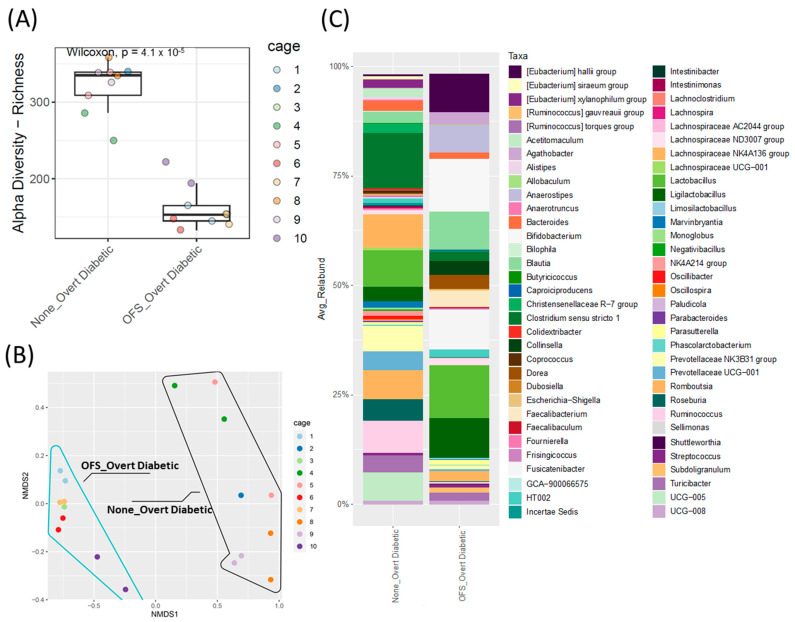
Cecal microbiota analysis of overt diabetic ZDSD rodents following OFS treatment. (**A**) Alpha diversity index, ASV/Species Richness with statistical significance between groups tested using pairwise Wilcoxon signed-rank test with FDR as a multiple hypothesis testing correction. (**B**) Beta-diversity, non-metric multidimensional scaling (NMDS) analysis based on Bray–Curtis dissimilarities; shape outlines used to represent sample groups with (blue line) or without (black line) OFS treatment. Statistical significance between groups was assessed using permutational multivariate analysis of variance using distance matrices (Adonis test). (**C**) Taxonomic analysis of genus-level relative abundance for control and OFS-treated ZDSD rodents (*n* = 18, genera with relative abundance lower than 0.1% were removed for visualization only).

Oligofructose reduced cecal microbiota alpha diversity and shifted beta diversity after 16 weeks of treatment (Figure 6A,B). The cecal, rather than fecal, microbiota was analyzed at the end of the study, as the cecum houses the majority of the gut microbiota and provides a more accurate endogenous representation of the proportional differences between genera [32]. OFS treatment also significantly shifted the relative abundance of the cecal microbiota with 17 taxa increased and only 1 taxon decreased (Supplementary Figure S4B). Similar to other OFS studies [33,34,35], the greatest increase observed was in *Bifidobacterium* relative abundance compared to the control untreated rats (Figure 6C and Figure S4B), while *Ruminococcus* relative abundance was significantly decreased. Of note, this was the only genus that was significantly altered from the genera in Figure 3 that were selected based on either potentially being causative or resultant of the diabetic phenotype.

## 4. Discussion

Rodent models are commonly used to examine the mechanism of T2D development and progression, although most rodent models fail to replicate the development and normal progression of diabetes that occurs in humans. In mice, long-term HFD-feeding causes weight gain and hyperglycemia associated with increased insulin and impaired glucose tolerance and is commonly used in the obese-susceptible C57BL/6J mice or SD rats [36,37]. However, these HFD-fed rodents do not develop overt T2D and fail to recapitulate the entire progression of human diabetes. Beta cell dysfunction is a characteristic of overt diabetes, and although a combination of HF-feeding and streptozotocin injection, to promote insulin resistance and destroy pancreatic beta cells, respectively, is another commonly used model of T2D, it fails to follow the natural progression of the disease unlike the ZDSD rat [38]. Furthermore, the genetic component of T2D development in humans is highly complex, with the genetic risk of diabetes development linked to multiple gene regions and gene interactions, making replication in rodents unachievable [2]. While the monogenic ZDF rodent model rapidly develops diabetes by 8 weeks of age due to a missense mutation in their leptin receptor, the ZDSD rat overcomes this by exhibiting normal leptin signaling and likely has polygenetic contributions to the development of diabetes. Additionally, the ZDF rat rapidly develops overt diabetes within 8 weeks of age, unlike the ZDSD model, which we and others have found provides a longer window to examine the progression of T2D and possible preventative therapeutics [9,11,39]. While the polygenic Otsuka Long-Evans Tokushima Fat rat (OLETF) also exhibits a prediabetic phase similar to the ZDSD rat, OLETF rats develop diabetes due to a lack of the cholecystokinin (CCK)-1 receptor, leading to hyperphagia and increased glucose production. However, these rats also have altered neuronal anatomy and melanocortin signaling, which does not occur in humans [40]. In the current study, rats progressed from a healthy to prediabetic phenotype by 16 weeks of age and developed diabetes by 24 weeks, with severe impairments in insulin production. This study supports the ZDSD model as a more relevant and translational model for the progression of diabetes. Similar to previous studies, we observed that blood glucose drastically increases, paralleled by increasing circulating insulin to attempt to compensate for the persistent hyperglycemia as the rats progress from a healthy to prediabetic phenotype. This is followed by a sharp decline in circulating insulin, representative of beta cell burnout as the rats progress into a diabetic state. Interestingly, these phenotypic changes are also paralleled by shifts in the gut microbiota.

T2D is associated with shifts in the relative abundance of specific bacterial genera and species [6,41]. However, most work examining the contribution of the gut microbiota to the development of T2D and vice versa is correlative, in part due to a lack of translational animal models that reflect T2D development in humans. Here, we demonstrate that shifts in both alpha and beta diversity, as well as specific bacterial genera, occur throughout the progression of T2D in ZDSD rats. We observed no significant changes in beta diversity from the healthy to prediabetic state, but following development of T2D, beta diversity was significantly shifted. Alpha diversity, which usually decreases with development of T2D, was increased in the prediabetic state. As prediabetes is a transitional state, the observed increase could be due to changes in metabolism or other host factors, driving changes in different bacterial species, which may warrant further investigation. We observed significant changes in specific bacterial genera both at the prediabetic and diabetic timepoints, indicating possible causative shifts driving the diabetic state or changes that might be secondary to the overt diabetic phenotype, respectively. In line with this, studies suggest that changes in the gut microbiota from HFD-feeding could drive the development of diabetes via increased metabolic endotoxemia, alterations in energy extraction, or changes in gut-derived metabolites, all of which could impact host physiological pathways that regulate glucose homeostasis [7,42,43]. Alternatively, hyperglycemia is known to impair gut barrier function, allowing for increased bacterial translocation and significantly shifts the gut microbiota, which is restored following a return to normoglycemia via insulin [44]. Therefore, whether these shifts in the diabetic state serve as a marker for disease progression via hyperglycemia or in a more causal role for disease development is unclear and warrants further investigation. In examining specific changes in bacterial genera and species over the progression from healthy to prediabetic and overt diabetic in the ZDSD rats, we may be able to better discriminate bacteria that serve as markers for the progression of T2D or that may play a causal role in driving the disease. These longitudinal shifts are likely not due to changes in diet or aging, as the HFD provided for 3 weeks was utilized to sync the development of diabetes between rats, which were then promptly returned to their normal chow diet. Although diet is known to shift the gut microbiota, these shifts are transient and are known to shift back to baseline after returning to a normal diet [45]. While aging is also known to alter the gut microbiota, significant shifts have not been observed in SD rats during the time course of our study [46]. Thus, we do not believe these shifts are merely an artifact of aging or a transient shift in diet but are a representation of the metabolic shifts of the ZDSD rats. However, it should be noted that this is one disadvantage of the ZDSD rodent model as it lacks a proper control to thoroughly address this. Nonetheless, in the present study, we found significant shifts in *Lactobacillus*, *Alistipes*, and *Ruminococcus* between the healthy and prediabetic state, indicating that these bacteria may participate in a more causal role, as these shifts occur prior to significant physiological changes such as overt hyperglycemia and hyperinsulinemia. The significant reductions in *Lactobacillus* during the development of T2D in the ZDSD rats highlights a possible protective role for this genus in the disease. As commonly used probiotics, several species of *Lactobacillus* are known to improve glycemia in both rodents and humans [47,48,49]. Conversely, the significant increases in the *Alistipes* and *Ruminococcus* genera indicate their presence may act to drive the development of T2D. While *Alistipes* relative abundance is increased in prediabetic and diabetic patients and in hyperglycemic mice, a causal role for increased *Alistipes* in T2D has not been established [50,51,52]. *Ruminococcus* is also increased in prediabetic and diabetic patients, and although its role in diabetes development is unclear, increases in *Ruminococcus* are associated with increased intestinal inflammation which could lead to gut barrier dysfunction and further metabolic consequences, potentially initiating disease development [53,54,55]. Conversely, the genera *Blautia*, *Lachnospiraceae UCG-001*, and *Roseburia* were shifted in only the diabetic state, indicating that host physiological changes may be influencing their relative abundance, highlighting their potential to serve as markers for T2D. *Blautia* is commonly increased in the microbiota of patients with T2D, and all *Blautia* strains can utilize glucose as a substrate to produce metabolites such as acetate, succinate, and lactate, providing a possible explanation for its bloom following diabetes development, when glucose is abundantly available [56,57,58]. *Lachnospiraceae UCG-001* and *Roseburia* are also short chain fatty acid (SCFA) producers but are commonly reduced with T2D [54,57]. While increases in *Lachnospiraceae UCG-001* are associated with improved weight loss in humans, no studies have examined their role in patients with diabetes [59]. *Roseburia* is a key producer of the SCFA, butyrate, and both *Roseburia* and butyrate can reduce intestinal inflammation and improve the gut barrier [54,60,61]. Thus, loss of *Roseburia* with T2D could further exacerbate the disease via increases in intestinal permeability and systemic inflammation. Overall, the changes in the bacterial genera observed over the course of the study reflect shifts previously observed in humans with prediabetes and T2D, supporting the ZDSD rat as a more translational model for T2D research from both a pathophysiological as well as microbial standpoint.

In further support of the ZDSD rat as a translational model for T2D, we observed significant shifts in fecal SCFA levels from the healthy to diabetic phenotype. Although shifts in fecal and circulating SCFA levels vary between studies, generally, increases in acetate and decreases in butyrate are associated with impairments in energy and glucose metabolism [22,62,63]. We observed a significant increase in fecal acetate in diabetic ZDSD rats, which was associated with increased fasting glucose and decreased fed and fasting insulin. This supports previous findings in rodents and humans, as circulating and fecal acetate levels have been shown to be increased in both rodents and patients with diabetes [62,64]. Although not fully understood, this is thought to be due to a reduction in acetate metabolism with T2D [65]. Furthermore, fecal acetate was positively correlated with several acetate producing bacteria—*Blautia*, *Fusicatenibacter*, and *Phascolarctobacterium. Blautia* was significantly increased only in diabetic ZDSD rodents, further highlighting its potential to serve as a marker for T2D. Additionally, increased acetate in the diabetic state could be a result of decreases in *Lachnospiraceae* and *Roseburia*, both of which can utilize acetate to produce butyrate [66]. While we observed no changes in fecal butyrate, fecal propionate was significantly increased in diabetic ZDSD rats and also associated with increased fasting glucose and decreased fed and fasting insulin. Increases in fecal propionate are associated with impairments in glycemia as well as increased T2D risk [63]. Additionally, fecal propionate has been found to be increased in patients with metabolic syndrome compared to healthy individuals [63,67,68]. However, as the fecal SCFA profile reflects the net result of SCFA production, uptake, and tissue utilization, we cannot determine whether the observed shifts are a direct result of microbial fermentation or due to the altered physiological state of the diabetic ZDSD rat, which remains to be explored. Regardless, the similarities between the SCFA profile of the ZDSD rat and humans with metabolic disorders further highlights the translational potential of this model in studying shifts in the microbiome and metabolome throughout the progression of T2D.

In order to examine the efficacy of using the ZDSD rat in the testing of potential therapeutic treatments for T2D, we supplemented their diet with 10% OFS in drinking water. While OFS treatment was unable to fully rescue the severely impaired glucose homeostasis in the overt diabetic ZDSD rodents, it did increase bodyweight and adiposity. This is contrary to findings in HFD-fed obese and insulin resistant rodents, where OFS is known to reduce food intake and bodyweight [22,69]. However, this discrepancy is likely due to the diabetic state of the rat; unlike SD rats that continue to gain weight when on a HF diet, body weight of the ZDSD rats declined during overt diabetes. To date, no studies have examined the effect of oligofructose treatment in an overt diabetic rodent model. We found that OFS treatment normalized bodyweight in the ZDSD rat, whereas untreated rats continued to lose weight. Insulin resistance combined with beta cell burnout prevents diabetic subjects from utilizing glucose, resulting in increased fat store utilization for energy. As such, we observed significant increases in adiposity in OFS-treated ZDSD rats, indicating that they may be better able to utilize glucose for energy. This increase in adiposity was paralleled by increased circulating leptin, which can improve insulin resistance and dysglycemia [70], which is possibly responsible for the observed improved glucose tolerance in the ZDSD rats after 15 weeks of OFS treatment. Although we only observed a trend in increased GLP-1 following OFS, samples were obtained during a fasted state, whereas GLP-1 is normally secreted following a meal. We did observe significant increases in *Gcg* mRNA in the LSI and colon of OFS-treated rats, which encodes for GLP-1, thus, we expect that, similar to previous studies, OFS supplementation increases postprandial GLP-1 levels, potentially improving glucose homeostasis. In line with this, we observed improvements in fed, but not fasted, glucose levels following OFS treatment, further supporting GLP-1 as a potential mediator of the improvements in glucose homeostasis. However, improvements in glucose homeostasis could also be due to improvements in insulin sensitivity, potentially via improvements in the gut barrier and metabolic endotoxemia. Shifts in the gut microbiota and increases in *Bifidobacterium* with OFS treatment has been shown to improve the gut barrier and decrease circulating LPS and metabolic endotoxemia [34,71]. We observed a significant increase in *Bifidobacterium* following treatment with OFS in the overt diabetic ZDSD model, which also exhibited improved glucose homeostasis. *Bifidobacterium* most commonly produce acetate and lactate and are commonly involved in cross-feeding with other beneficial bacteria such as the butyrate-producing *Faecalibacterium prausnitzii* and *Roseburia* [72,73]. Increases in these bacteria and butyrate could improve the gut barrier’s integrity, decreasing systemic inflammation to reduce insulin resistance and improve glycemia [74]. Although we examined cecal microbiota, while OFS treatment improved glucose homeostasis in diabetic ZDSD rats, it did not seem to impact many of the genera that were differentially altered in the prediabetic or diabetic state. Only *Ruminococcus*, which was increased in prediabetic and diabetic ZDSD fecal microbiota, was reduced following OFS treatment, further highlighting the potential significance of *Ruminococcus* in glucose homeostasis. Overall, the slight improvements in glucose homeostasis following OFS highlight the efficacy of the ZDSD model in testing potential treatments for T2D.

## 5. Conclusions

Our findings confirm the ZDSD model as a translational model for T2D and establish a role for the gut microbiota in the development and progression of the disease. The progression from healthy to prediabetic to diabetic states in the ZDSD rat is paralleled by shifts in specific bacterial genera that could serve as markers for T2D or indicate a causal role for the gut microbiota in the progression of the disease. These shifts are associated with alterations in fecal SCFA levels as well as metabolic parameters. Furthermore, treatment with OFS was able to improve glucose tolerance in the overt diabetic ZDSD rat, highlighting its potential for use in the evaluation of therapeutics targeting the gut microbiota.

## Data Availability

All data generated or analyzed during this study are included in this published article and its supplementary information files.

## Supplementary Materials

The following supporting information can be downloaded at: https://www.mdpi.com/article/10.3390/metabo13050660/s1, Figure S1: Body composition at 8, 16, and 24 weeks in the ZDSD rodent model. (A) Fat and lean mass of ZDSD rats. Data in all graphs represent the mean + SEM (*n* = 18); * *p* < 0.05, *** *p* < 0.001 vs. 8 weeks. ### *p* < 0.001 vs. 16 weeks. Figure S2: Differential expression analysis of taxonomic shifts in (A) Diabetic vs. Healthy and (B) Diabetic vs. Prediabetic fecal samples. Diabetic group is a baseline. Taxa showed as “increased” on the graph are decreased in Diabetic group compared to Healthy (A) or Prediabetic (B) groups. Figure S3: Time course analysis of fecal short chain fatty acids (SCFAs) throughout the development of diabetes in ZDSD rodents. (A) Isobutyric acid, (B) isovaleric acid, (C) valeric acid, and (D) hexanoic acid levels in the feces of 5 hour fasted ZDSD rats. Data in all graphs represent the mean + SEM (*n* = 18); * *p* < 0.05, *** *p* < 0.001, as assessed by Kruskal–Wallis one-way ANOVA. Figure S4: (A) Fat and lean mass of OFS-treated or untreated ZDSD rats. (B)Differential expression analysis of taxonomic shifts in Overt Diabetic vs. OFS-treated Overt Diabetic cecal samples, where untreated Overt Diabetic was used as a baseline. Data in all graphs represent the mean + SEM (*n* = 18); *** *p* < 0.001 vs. control.
