# Supplementary material for: Longitudinal Characterization of the Gut Microbiota in the Diabetic ZDSD Rat Model and Therapeutic Potential of Oligofructose
Source: Metabolites. 2023 May 16;13(5):660. doi: 10.3390/metabo13050660 (PMC10220957; doi:10.3390/metabo13050660)
Supplement: Supplementary file 1 [file metabolites-13-00660-s001.zip › metabolites-2325284-supplementary.pdf]

(A)

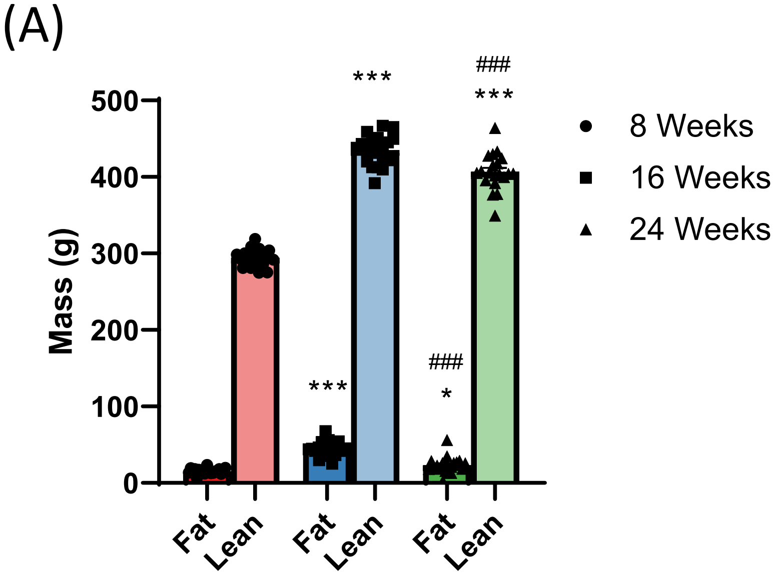

Supplementary Figure S1: Body composition at 8, 16, and 24 weeks in the ZDSD rodent model. (A) Fat and lean mass of ZDSD rats. Data in all graphs represent the mean + SEM (n=18); *p < 0.05, ***p < 0.001 vs 8 weeks. ###*p* < 0.001 vs 16 weeks.

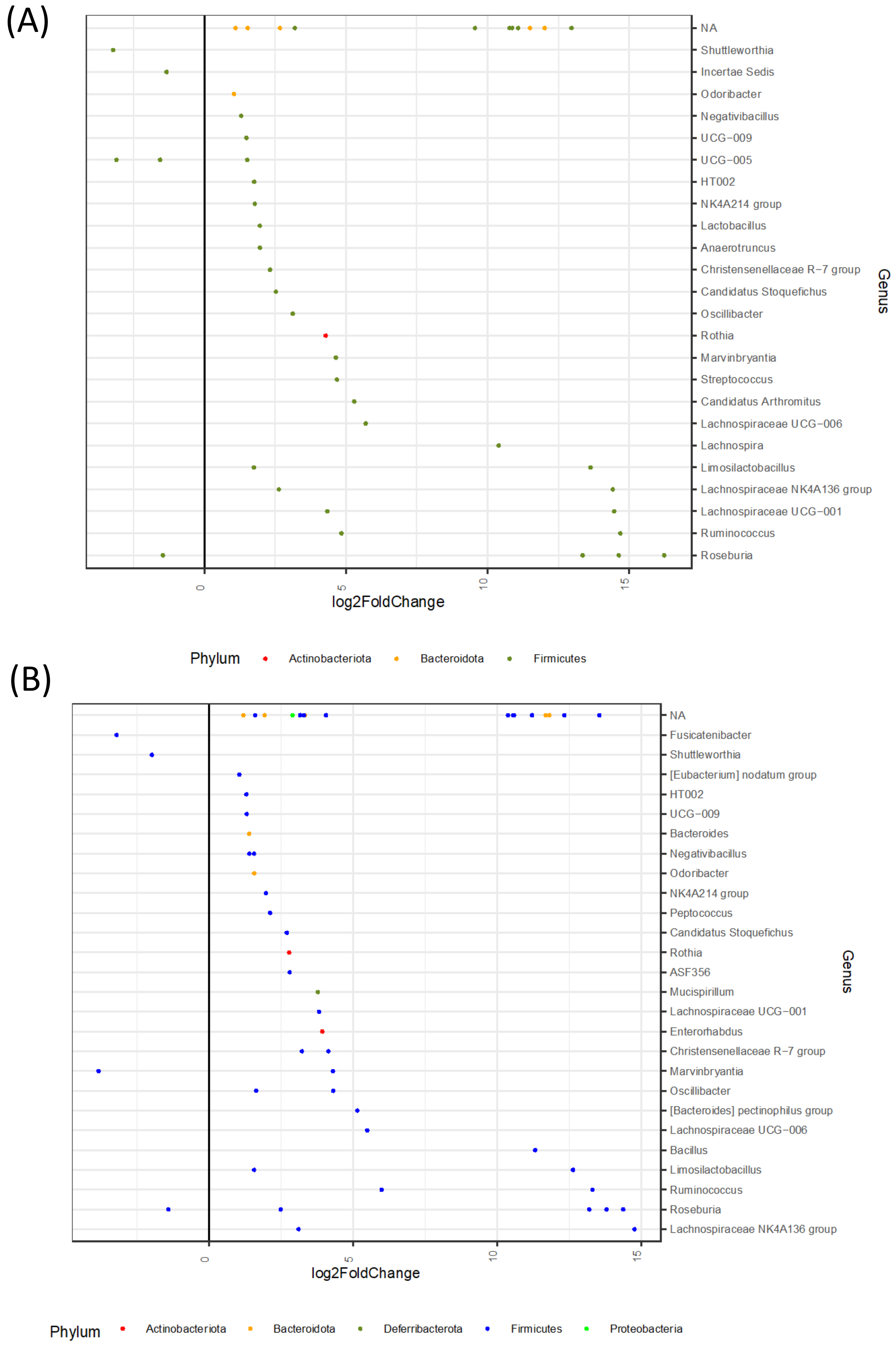

Supplementary Figure S2: Differential expression analysis of taxonomic shifts in (A) Diabetic vs Healthy and (B) Diabetic vs Prediabetic fecal samples. Diabetic group is a baseline. Taxa showed as “increased” on the graph are decreased in Diabetic group compared to Healthy (A) or Prediabetic (B) groups.

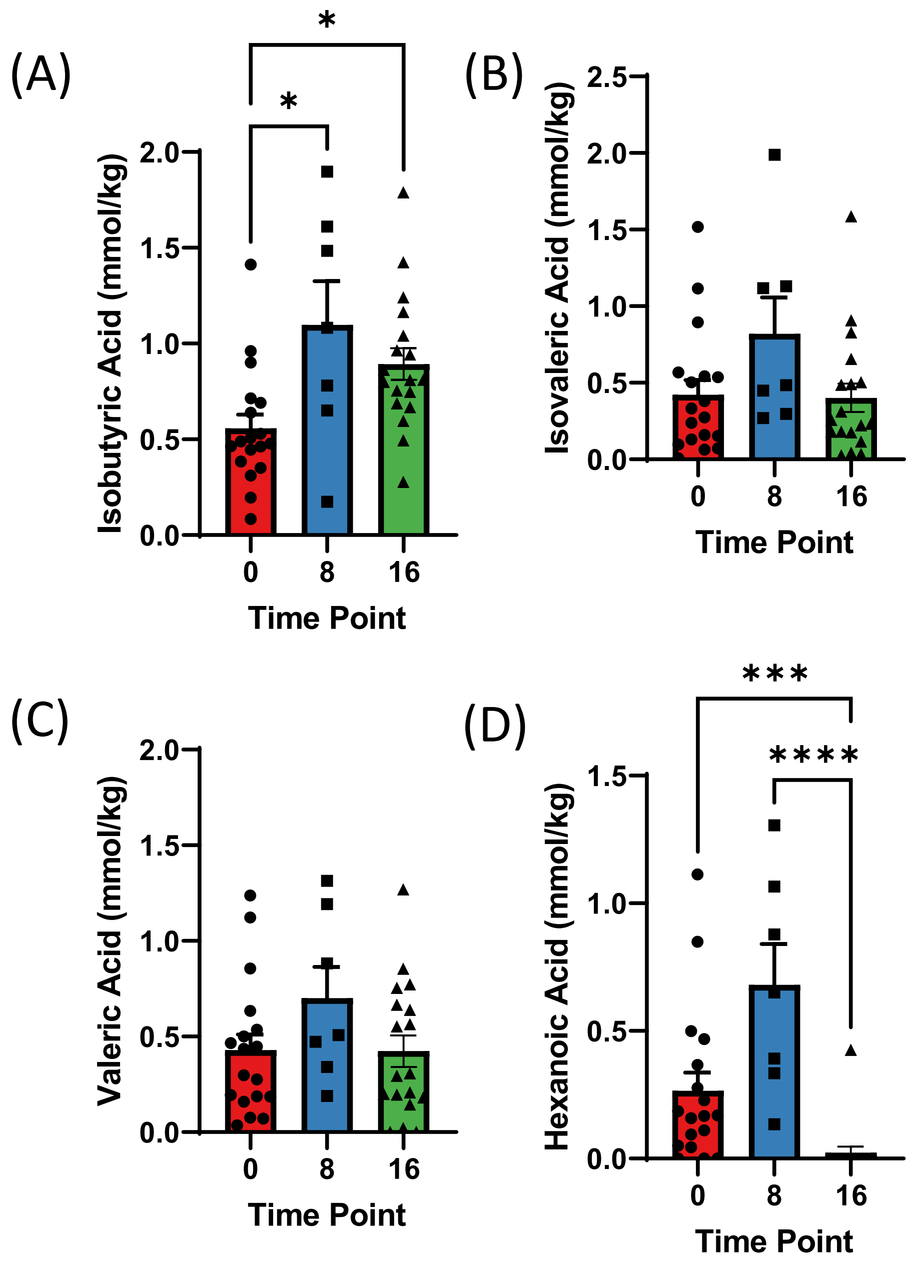

Supplementary Figure S3: Time course analysis of fecal short chain fatty acids (SCFAs) throughout the development of diabetes in ZDSD rodents. (A) Isobutyric acid, (B) isovaleric acid, (C) valeric acid, and (D) hexanoic acid levels in the feces of 5 hour fasted ZDSD rats. Data in all graphs represent the mean + SEM (n=18); *p < 0.05, ***p < 0.001, **** is p < 0.0001 as assessed by Kruskal–Wallis one-way ANOVA.

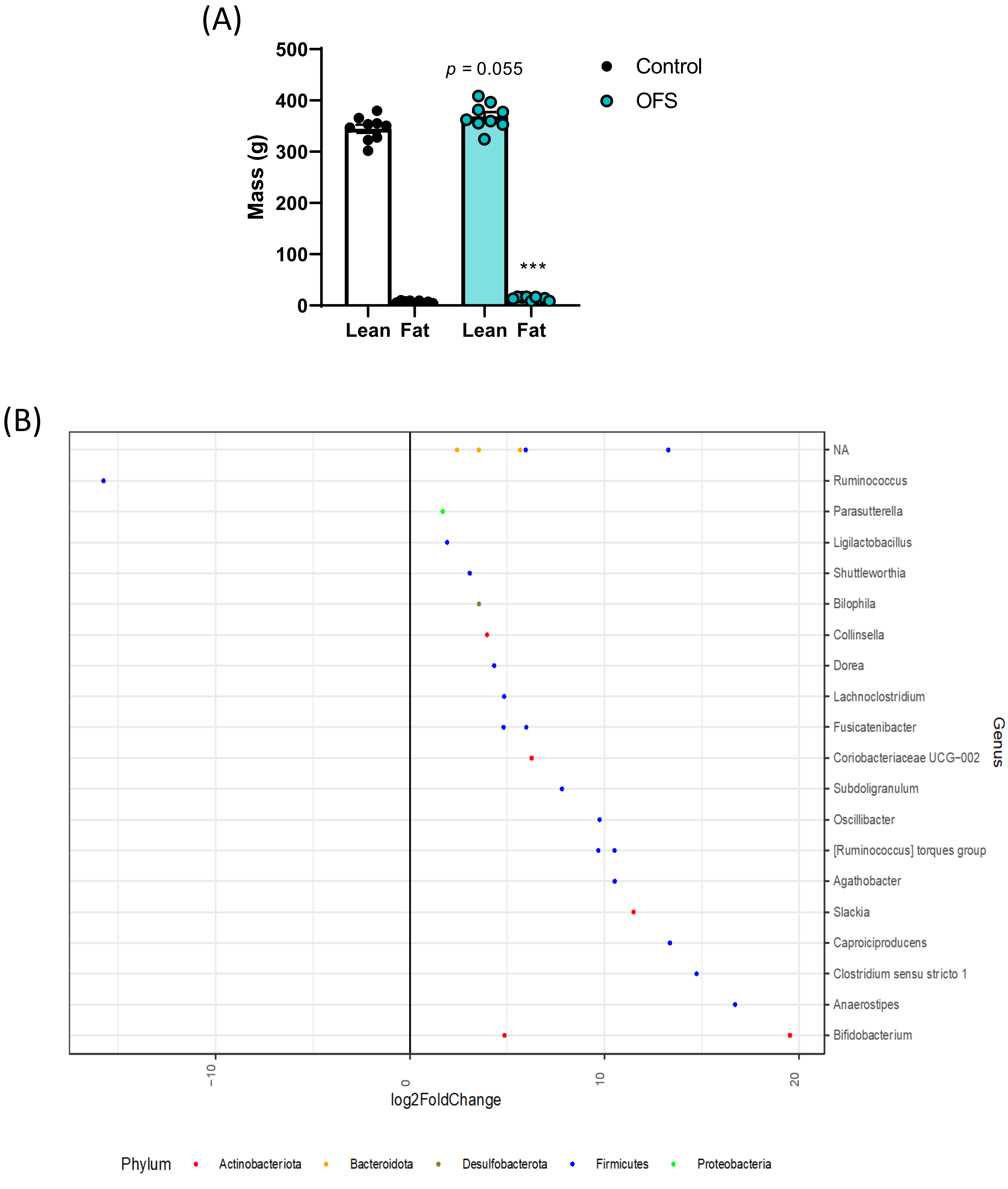

Supplementary Figure S4: (A) Fat and lean mass of OFS-treated or untreated ZDSD rats. (B) Differential expression analysis of taxonomic shifts in Overt Diabetic vs OFS-treated Overt Diabetic cecal samples, where untreated Overt Diabetic was used as a baseline. Data in all graphs represent the mean + SEM (n=18); ***p < 0.001 vs control.
